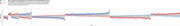# Distinct CSF proteomic signatures are associated with cognitive decline in A+T+ and A+T‐ individuals with MCI due to Alzheimer’s Disease

**DOI:** 10.1002/alz.090645

**Published:** 2025-01-09

**Authors:** Eleonora M. Vromen, Argonde C. van Harten, Charlotte Teunissen, Wiesje M. van der Flier, Yolande A.L. Pijnenburg, Pieter Jelle Visser, Betty M. Tijms

**Affiliations:** ^1^ Alzheimer Center Amsterdam, Neurology, Vrije Universiteit Amsterdam, Amsterdam UMC location VUmc, Amsterdam Netherlands; ^2^ Amsterdam Neuroscience, Neurodegeneration, Amsterdam Netherlands; ^3^ Amsterdam UMC, Amsterdam Netherlands; ^4^ Epidemiology and Data Science, Vrije Universiteit Amsterdam, Amsterdam UMC location VUmc, Amsterdam, Noord‐Holland Netherlands; ^5^ Alzheimer Center Amsterdam, Neurology, Vrije Universiteit Amsterdam, Amsterdam UMC, Amsterdam Netherlands; ^6^ Amsterdam Neuroscience, Neurodegeneration, Amsterdam, Noord‐Holland Netherlands; ^7^ Department of Psychiatry and Neuropsychology, School for Mental Health and Neuroscience, Maastricht University, Maastricht Netherlands; ^8^ Department of Neurobiology, Care Sciences and Society, Division of Neurogeriatrics, Karolinska Institutet, Stockholm Sweden

## Abstract

**Background:**

Individuals with mild cognitive impairment (MCI) due to Alzheimer’s disease (AD) show variability in cognitive decline. Little is known about the underlying mechanisms, but these are likely to depend on tau levels. Using untargeted proteomics in cerebrospinal fluid (CSF), we studied which processes were associated with cognitive decline in A+T‐ and A+T+ MCI individuals.

**Method:**

We included 80 individuals from ADC (age 66±7.9 years, 28 (35%) women, 52 (66%) A+T+, 4.6±2 follow‐ups over 4.1±1.8 years). N=2323 CSF proteins were quantified at baseline using untargeted TMT LC‐MS/MS. We tested associations between each protein and 1) progression to dementia with Cox proportional hazard models and 2) decline over time on MMSE using linear mixed models with subject‐specific intercepts. All analyses were stratified for tau status and adjusted for age, sex and education. We performed enrichment analysis on proteins associated with cognitive decline.

**Result:**

Both AT groups declined over time on MMSE, with steeper decline in A+T+ (A+T+ β±SE ‐0.99±0.08 p‐value<0.001, A+T‐ β±SE ‐0.39±0.09 p‐value<0.001, p‐value_A+T‐ vs. A+T+_<0.001). Largely different groups of proteins predicted faster progression to dementia and steeper decline on MMSE in A+T+ (resp. n=119 and n=664, p‐values<0.05, Figure 1) compared with A+T‐ individuals (resp. n=88 and n=718, p‐values<0.05). In A+T+ higher levels of proteins associated with synaptic plasticity processes and extracellular matrix organization, and lower levels of proteins associated with immune system processes and lipid remodeling were associated with steeper cognitive decline. Conversely, in A+T‐ these processes were associated with slower cognitive decline. Furthermore, in A+T‐ proteins indicative of blood‐brain barrier (BBB) impairment were also associated with cognitive decline.

**Conclusion:**

A+T+ and A+T‐ individuals with MCI show largely different proteomic signatures associated with cognitive decline. In A+T+ MCI individuals, higher levels of synaptic‐plasticity proteins and lower levels of immune‐related proteins were associated with faster cognitive decline, while in A+T‐ opposite effects were found. This suggest that treatments to slow cognitive decline in AD may need tailoring on tau status.